# Nursing perception and practices in N95 mask reuse in intensive care: a cross-sectional study

**DOI:** 10.1590/0034-7167-2025-0261

**Published:** 2026-06-22

**Authors:** Viviane Lopes Vimieiro, Claysson Bruno Santos Vimieiro, Adriana Cristina Oliveira

**Affiliations:** I Universidade Federal de Minas Gerais. Belo Horizonte, Minas Gerais, Brazil; II Pontifícia Universidade Católica de Minas Gerais. Belo Horizonte, Minas Gerais, Brazil

**Keywords:** N95 Respirators, Health Personnel, Occupational Health, Intensive Care Units, Nursing., Respiradores N95, Personal de Salud, Salud Laboral, Unidades de Cuidados Intensivos, Enfermería.

## Abstract

**Objectives:**

to analyze Intensive Care Unit nursing professionals’ perceptions and practices regarding safe N95 mask use in reuse protocols.

**Methods:**

a cross-sectional study conducted in Intensive Care Units.

**Results:**

two hundred sixty-five professionals participated. Most did not feel safe with the adopted protocol. Their main concerns were humidity, dirt, and storage. Less than 1% mentioned filtration efficiency or training. Fifty-three point six percent (seven days) and 74.1% (15 days) received training, primarily in 2020. Donning and doffing were the most frequently discussed topics. Seal verifications and visual inspections were always performed by 20.9% and 46.4% (seven days), and by 26.8% and 35.7% (15 days), respectively. There was a significant association between training and seal verification in the seven-day protocol. All reported a lack of dedicated storage space for N95 masks.

**Conclusions:**

the perception of safety was weak, and training for safe reuse was insufficient.

## RESUMEN

**Objetivos:**

analizar las percepciones y prácticas del personal de enfermería de unidades de cuidados intensivos respecto al uso seguro de mascarillas N95 en protocolos de reutilización.

**Métodos:**

estudio transversal realizado en unidades de cuidados intensivos.

**Resultados:**

participaron 265 profesionales. La mayoría no se sentía segura con el protocolo adoptado. Sus principales preocupaciones eran la humedad, la suciedad y el almacenamiento. Menos del 1% mencionó la eficiencia de la filtración o la capacitación. El 53,6% (siete días) y el 74,1% (15 días) recibieron capacitación, principalmente en 2020. Vestirse y desvestirse fue el tema más discutido. El 20,9% y el 46,4% (siete días) y el 26,8% y el 35,7% (15 días) realizaron siempre las comprobaciones de precinto y las inspecciones visuales, respectivamente. Se observó una asociación significativa entre la capacitación y la verificación del sello en el protocolo de siete días. Todos informaron falta de espacio de almacenamiento específico para mascarillas N95.

**Conclusiones:**

la percepción de seguridad fue deficiente y la formación para la reutilización segura fue insuficiente.

## INTRODUCTION

Personal protective equipment (PPE) use is an essential measure for the occupational safety of healthcare professionals^(1)^. Among these devices, the N95 mask stands out as one of the main barriers against respiratory microorganisms, such as *Mycobacterium tuberculosis*, the influenza virus, and Severe Acute Respiratory Syndrome Coronavirus 2 (SARS-CoV-2), the causative agent of coronavirus disease 2019 (COVID-19)^(1-3)^.

High-impact epidemiological events, such as the SARS epidemic in 2003, the H1N1 influenza pandemic in 2009, the emergence of Middle East Respiratory Syndrome Coronavirus (MERS-CoV) in 2012, and, more recently, the COVID-19 pandemic in 2020, have highlighted the vulnerability of healthcare systems to global health crises. These contexts have resulted in a global shortage of N95 masks, prompting the adoption of alternative strategies, such as extended use and reuse of these devices^(4-7)^.

N95 masks are designed to ensure a minimum filtration efficiency of 95% for particles up to 0.3 micrometers (µm), in addition to ensuring a proper facial seal on the users’ face^(2,3)^. The manufacture and certification of these devices are regulated by national and international standards, such as the Brazilian Association of Technical Standards (In Portuguese, *Associação Brasileira de Normas Técnicas* - ABNT) NBR 13698:2011, in Brazil, the European Standard (EN) 149:2001+A1:2009, in Europe, and the Code of Federal Regulations (CFR) 42 Part 84 of the National Institute for Occupational Safety and Health, in the United States, which establish performance and safety criteria^(8-10)^.

In healthcare institutions, especially those with high PPE demands, such as Intensive Care Units (ICUs), reusing these devices has been adopted as an institutional strategy to rationalize resources. While practical in terms of supply, this practice poses significant challenges to worker protection. Reuse favors device structural degradation, with potential damage to the face seal due to fastening strap wear and nose clip deformation^(11,12)^. Furthermore, the lack of adequate storage facilities is a critical factor, as it can contribute to cross-contamination and loss of integrity of N95 masks^(11,12)^.

In this context, nursing professionals stand out because they constitute the largest contingent of the multidisciplinary team and because they spend long periods in direct contact with patients. They are, therefore, among the most vulnerable to exposure to respiratory agents^(13-15)^. These professionals are responsible for correctly applying institutional protocols and adopting safe practices when handling N95 masks, such as visual inspection, seal verification by exhaling and inhaling, and proper storage. Evidence indicates that adherence to these practices is associated with access to specific and up-to-date training that covers essential topics, such as donning and doffing, seal verification, N95 mask integrity assessment, hand hygiene, disposal criteria, and appropriate storage practices^(2,3,16)^.

Despite the relevance of the topic, there is still little evidence that comprehensively explores nursing professionals’ perceptions and practices regarding N95 mask reuse protocols, especially in highly complex care settings such as ICUs. Therefore, it is crucial to understand how these professionals assess the protocols, what practices they adopt based on the guidance they receive, and how training influences adherence to safety measures. This analysis helps inform the improvement of institutional strategies, strengthen an evidence-based safety culture, and ensure adequate protective conditions in critical care settings.

## OBJECTIVES

To analyze ICU nursing professionals’ perception and practices regarding safe N95 mask use in reuse protocols.

## METHODS

### Ethical aspects

The study was conducted in accordance with the ethical principles of Resolution 466/2012 of the Brazilian National Health Council regarding research with human beings, and was approved by the *Universidade Federal de Minas Gerais* Research Ethics Committee, whose opinion is attached to this submission.

### Study design, period, and location

This is a cross-sectional study, developed through interviews with nursing professionals, using a structured questionnaire and guided by the STrengthening the Reporting of OBservational studies in Epidemiology tool^(17)^. Data collection took place from December 2023 to August 2024 in adult ICUs of two hospitals located in Belo Horizonte, Minas Gerais, Brazil. To preserve anonymity, each unit was identified with the codes UA, UB, UC, and UD. The UA and UB ICUs belong to a private institution and serve private or insured patients with 40 general ICU beds and 20 cardiovascular ICU beds. The UC and UD ICUs are part of a public university institution, which exclusively assists Brazilian Health System users, with 18 intensive care beds and 19 coronary care unit beds.

### Population or sample; inclusion and exclusion criteria

The convenience sample consisted of nursing professionals working directly in the selected ICUs, using N95 masks according to reuse protocols at sevenor 15-day intervals. According to institutional protocol, replacement could occur earlier than the established deadline in cases of dirt, humidity, structural damage, fixing strap or nose clip rupture, loss of seal, or contamination by blood, body fluids, and respiratory/nasal secretions. Nursing professionals who did not comply with these intervals, were on vacation, sick leave, or maternity leave, or who refused to respond to the questionnaire were excluded.

The sample size calculation was based on the finite population proportion estimation method, considering 377 eligible nursing professionals. An estimated proportion of 50% (p=0.5), a 5% margin of error, and a 95% confidence level were adopted, resulting in a minimum sample of 191 participants. However, the final sample consisted of 265 professionals.

### Study protocol

Data were collected through a structured questionnaire developed by the researcher based on guidelines from the Center for Disease Control and Prevention and the Brazilian National Health Regulatory Agency. The instrument contained 24 questions organized into three sections: (i) sociodemographic, occupational, and educational variables; (ii) aspects related to the institutional protocol for reusing N95 masks and professionals’ perceptions of the safety provided. In this section, participants also indicated the aspects considered essential to ensure respiratory protection in reuse protocols, such as fastening element integrity, adequate sealing, presence of humidity or dirt, risk of contamination, training received, storage conditions, and durability and filtration efficiency; and (iii) participation in training, in addition to practice in seal verification, visual inspection, and N95 mask storage.

A pilot test was conducted with ICU nursing professionals with similar profiles to those of the study institutions to assess the clarity of the questions and the applicability of the instrument. Based on the observations from this stage, minor adjustments were made to the questionnaire.

Eligible nursing professionals were invited to participate in the study and informed about the research objectives, the voluntary nature of participation, and data confidentiality. After reading and signing the Informed Consent Form, the interviews were conducted in person by the researcher, individually and at times compatible with participants’ work routines. The mean time to complete the questionnaire was approximately ten minutes.

### Analysis of results and statistics

The collected data were stored in Microsoft Excel^®^ 2019 and subsequently analyzed in the Statistical Package for the Social Sciences version 28.0. In descriptive analysis, qualitative variables were described by absolute and relative frequencies. For quantitative variables, mean, standard deviation, and range measurements were used. To compare the relationship between training participation and the frequency with which nursing professionals adopted safety practices when using N95 masks, specifically in terms of seal verification and visual inspection, chi-square and Fisher’s exact tests were used. The significance level was set at 5% in all analyses, with a 95% Confidence Interval.

## RESULTS

A total of 377 nursing professionals were eligible for the study. In ICUs with a seven-day reuse protocol, 74.3% (n=153) of 206 eligible professionals participated; 8.7% (n=18) were excluded due to absence (vacation, sick leave, or maternity); and 17% (n=35) refused. In ICUs with a 15-day protocol, 65.5% (n=112) of 171 eligible professionals participated; 9.4% (n=16) were excluded due to absence; and 25.1% (n=43) refused. Therefore, the sample consisted of 268 (71.1%) professionals, and Table 1 presents the sociodemographic, work, and educational characteristics.

**Table 1:** Sociodemographic, occupational, and training characteristics of nursing professionals in Intensive Care Units who adopted seven-day (n=153) and 15-day (n=112) reuse protocols on N95 mask use, Belo Horizonte, Minas Gerais, Brazil, 2025

Variables	Seven-day protocol (n=153)		15-day protocol (n=112)	
	n	%	n	%
**Sex**				
Female	116	75.8	81	72.3
Male	37	24.2	31	27.7
**Professional category**				
Nurse	25	16.3	68	60.7
Nursing technician	128	83.7	44	39.3
**Degree**				
Graduation	10	40.0	8	11.8
Specialist	15	60.0	53	77.9
Master’s	0	0.0	6	8.8
Doctoral	0	0.0	1	1.5
**Age (years)** Mean (±SD/range)	39.4 (±8.9; 22-61)		40.8 (±6.6; 25-57)	
**Length of professional training (years)** Mean (±SD/range)	13.4 (±7.5; 2-34		15.5 (±5.8; 2-26)	
**Length of professional experience (years)** Mean (±SD/range)	13.2 (±7.5; 2-34)		15.2 (±6.0; 2-26)	
**Length of service at the institution (years)**				
Mean (±SD/range)	8.1 (±6.2; 1-34)		7.3 (±4.6;1-23)	
**Time working in the ICU (years)**				
Mean (±SD/range)	10.7 (±6.7; 1-34)		8.6 (±5.1; 1-24)	
**Work shift**				
Daytime	81	52.9	64	57.1
**Weekly working hours (hours)**				
≤36	19	12.4	104	92.9
≥40	134	87.6	8	7.1
**Performance in another institution**				
Yes	44	28.8	42	37.5
No	109	71.2	70	62.5

*SD - Standard Deviation; ICU - Intensive Care Unit.*

A predominance of female nursing professionals was observed in both protocols. In the seven-day protocol, the majority were nursing technicians (n=128; 83.7%), while in the 15-day protocol, nurses predominated (n=68; 60.7%). Qualifications also differed between the groups. In the 15-day protocol, there was a higher percentage of specialists regarding qualifications (n=53; 77.9%), including master’s degree (n=6; 8.8%) and doctoral degree (n=1; 1.5%), while in the seven-day protocol, the majority had only a bachelor’s degree (n=10; 40.0%) or specialization (n=15; 60.0%).

Two ICUs adopted a protocol for reusing N95 masks for up to seven consecutive shifts, and two others for up to 15 shifts, with shifts lasting six to 12 hours. In relation to nursing professionals’ perceptions of the safety of these protocols, 42.5% (n=65) of participants enrolled in the seven-day protocol considered it safe; 25.5% (n=39) classified it as unsafe; and 32% (n=49) did not know. In the 15-day protocol, 33% (n=37) considered the protocol safe; 25.9% (n=29) considered it unsafe; and 41.1% (n=46) did not know.

**Figure 1:**
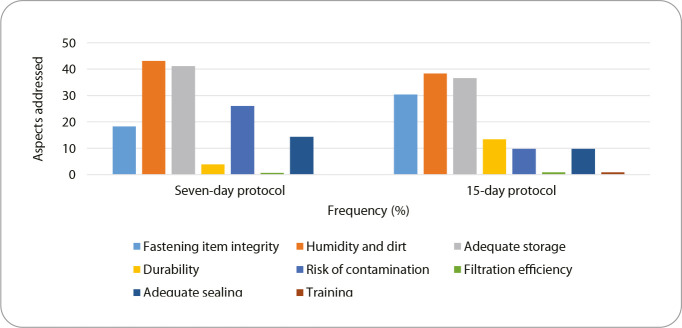
Respiratory protection perceived by nursing professionals in Intensive Care Units seven-day (n=153) and 15-day (n=112) reuse protocols on N95 mask use, Belo Horizonte, Minas Gerais, Brazil, 2025

Additionally, Figure 1 presents the aspects addressed by nursing professionals as fundamental to ensuring respiratory protection during N95 mask reuse protocols.

In the analysis of the sevenand 15-day N95 mask reuse protocol, aspects such as “humidity and dirt” and “safe storage conditions” stood out as points of concern among nursing professionals. In contrast, “ensuring minimum filtration capacity” was mentioned by only one professional (0.9%) in both protocols, while “completion of training” appeared only in the 15-day protocol, also mentioned by a single participant. These data indicate that, even with the possibility of multiple responses in the open-ended question, these criteria received little emphasis.

Concerning participation in training on N95 mask use, 53.6% (n=82) of nursing professionals in the seven-day protocol reported receiving training, while in the 15-day protocol, this percentage was 74.1% (n=83). For the majority, training occurred during the COVID-19 pandemic: 79.3% (n=65) in the seven-day group and 88% (n=73) in the 15-day group. In both protocols, the duration was less than two hours, and training predominantly took place at the institution itself. Table 2 details the data.

**Table 2:** Participation of nursing professionals from Intensive Care Units with seven-day (n=153) and 15-day (n=112) reuse protocols in training on N95 mask use, Belo Horizonte, Minas Gerais, Brazil, 2025

Variables	Seven-day protocol (n=153)		15-day protocol (n=112)	
	n	%	n	%
**Training participation**				
Yes	82	53.6	83	74.1
No	66	43.1	26	23.2
Do not know	5	3.3	3	2.7
**Year**				
2020	65	79.3	73	88.0
2020 and 2021	5	6.1	3	3.6
2021 2022 2023	8 3 1	9.7 3.7 1.2	4 2 1	4.8 2.4 1.2
**Duration**				
Less than two hours	69	84.2	77	98.2
More than two hours	12	14.6	3	3.6
Unable to say	1	1.2	3	3.6
**Location**				
At the institution itself	70	85.4	61	73.5
At another institution	3	3.6	10	12.0
At both locations	9	11.0	12	14.5

**Figure 2:**
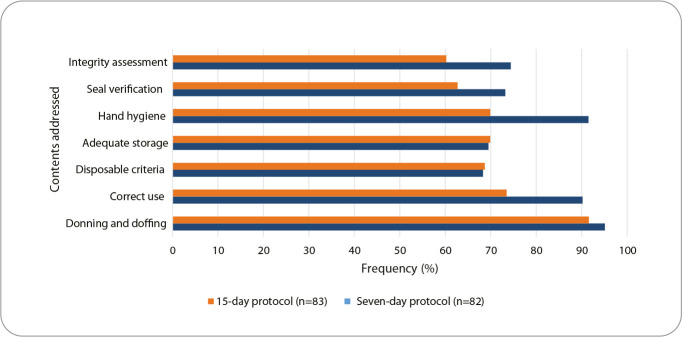
Contents covered in training for nursing professionals in Intensive Care Units with seven-day (n=153) and 15-day (n=112) reuse protocols on N95 mask use, Belo Horizonte, Minas Gerais, Brazil, 2025

In the context of the content covered in training on N95 mask use, Figure 2 details the topics covered during the training.

“Donning and doffing” was the most frequently discussed topic in both protocols, by 77 (92.8%) professionals in the 15-day protocol and 75 (91.5%) professionals in the seven-day protocol. In the seven-day protocol, “correct and safe use” (72; 87.8%) and “hand hygiene” (74; 90.2%) stood out. The least frequently discussed topics were “integrity assessment” (54; 65.1%) and “seal verification” (56; 67.4%) in the 15-day protocol, and “safe storage” (58; 70.7%) and “disposal criteria” (60; 73.2%) in the seven-day protocol.

Concerning the adoption of safe practices, in the seven-day protocol, seal verification was rarely performed by 45.8% (n=70), always by 20.9% (n=32), never by 19.6% (n=30), and was unknown by 13.7% (n=21). In the 15-day protocol, the percentages were 50.0% (n=56) reported performing the practice rarely, 26.8% (n=30) always, 17.9% (n=20) never, and 5.4% (n=6) were unaware of this step. As for visual inspection, in the seven-day protocol, 47.7% (n=73) reported performing it rarely, 46.4% (n=71) always, and 5.9% (n=9) never. In the 15-day protocol, 60.7% (n=68) performed it rarely, 35.7% (n=40) always, and 3.6% (n=4) never. Table 3 presents the association between participation in training and adoption of practices related to seal verification and visual inspection of N95 masks.

**Table 3:** Training on N95 mask use and seal verification practices and visual inspection among nursing professionals in Intensive Care Units with seven-day (n=153) and 15-day (n=112) reuse protocols, Belo Horizonte, Minas Gerais, Brazil, 2025

Practices	Variables	Trainings				*p* value
		Yes (n=82)		No (n=66)		
		n	%	n	%	
**Seven-day protocol**						
**Seal verification**	Unknown	6	7.3	15	22.7	0.002^*^
	Never	13	15.9	17	25.8	
	Rarely	44	53.6	21	31.8	
	Always	19	23.2	13	19.7	
**Visual inspection**	Never	3	3.7	6	9.1	0.198^**^
	Rarely	35	42.6	37	56.1	
	Always	44	53.7	23	34.8	
**Practices**	**Variables**	**Trainings**				***p* value**
		**Yes (n=83)**		**No (n=29)**		
		**n**	**%**	**n**	**%**	
**15-day protocol**						
**Seal verification**	Unknown	3	3.6	3	10.3	0.374^**^
	Never	15	18.1	5	17.2	
	Rarely	42	50.6	14	48.3	
	Always	23	27.7	7	24.1	
**Visual inspection**	Never	2	2.4	2	6.9	0.298^**^
	Rarely	54	65.1	14	48.3	
	Always	27	32.5	13	44.8	

* Chi-square test;

** Fisher’s exact test.

The comparison between trained and untrained professionals revealed a statistically significant difference in seal verification only in the seven-day protocol (p=0.002), with greater adherence among those who participated in training. For visual inspection, no statistically significant differences were observed in either protocol, indicating that training did not influence the adoption of this practice.

In relation to storage location, all participants in both protocols (100%, n=153; n=112) reported a lack of dedicated storage space for N95 masks after use in the institutions where they worked. Given this limitation, participants adopted different storage strategies. In the seven-day protocol, most stored masks in plastic bags inside their purses or personal lockers (61.4%; n=94). In the 15-day protocol, the personal locker was the preferred storage location, used by 43.8% (n=49), while plastic bag use was less common (6.3%; n=7).

## DISCUSSION

The findings of this study highlight critical aspects related to ICU nursing professionals’ perception and practices regarding safe N95 mask use in reuse protocols. In these institutions, reuse is permitted for seven or 15 consecutive shifts, in sixor 12-hour shifts, as long as the N95 masks remain clean, intact, dry, and have an adequate face seal. The continuation of this measure, even after the end of the global public health emergency caused by COVID-19, reflects its consolidation as an institutional strategy for cost reduction.

However, these guidelines contrast with the recommendations of international and national regulatory bodies, which classify N95 masks as single-use devices, recommending their disposal after each use^(2,3)^. Technical standards in different countries establish strict parameters for filtration performance and efficiency, but do not include criteria for reuse, highlighting a regulatory gap^(8-10)^. Furthermore, variability is observed between countries and institutions: some regulations allow only extended use or limited reuse, while others combine both practices, resulting in different protocols^(6,18-20)^.

This heterogeneity highlights the lack of consensus on the safe reuse limits for N95 masks, which may compromise nursing professionals’ confidence in the adopted protocols. In this study, most participants reported not considering the seven-day (71.9%) or 15-day (60.7%) protocols safe, signaling a perception of insecurity even in the presence of locally defined protocols. These findings corroborate the literature, which indicates that the lack of clear and unified protocols contributes to healthcare worker insecurity, highlighting the need for consistent, evidence-based guidelines to ensure safe N95 mask use in healthcare settings^(21)^.

Nursing professionals identified humidity, dirt, and improper storage as the main risk factors for respiratory protection in the sevenand 15-day N95 mask reuse protocols. These factors reflect the team’s perceptions of relevant practices, as they compromise the structural integrity of N95 masks over time. During reuse, N95 masks tend to accumulate dirt on the outer and inner layers, as well as retain humidity, conditions aggravated by the lack of adequate storage space. These factors, combined, contribute to wear and tear on the device and can increase occupational risks for healthcare professionals^(22)^.

However, equally essential aspects of occupational safety in reuse protocols, such as maintaining filtration efficiency and conducting training, were mentioned by less than 1% of participants. This low frequency reveals a significant gap in perception regarding fundamental criteria regarding the device’s ability to maintain its effectiveness over time. Quasi-experimental research demonstrated that, after use for 14 consecutive shifts, the filtration efficiency of N95 masks reduced to 80.4%, compromising the protection offered to users^(23)^. Furthermore, the low value given to training suggests the need to reinforce, in training processes, the importance of continuous training as a strategy for safe PPE use.

Concerning participation in training on N95 mask use, a higher proportion of trained professionals was found in public institutions (74.1%), compared to private institutions (53.6%), in the 15-day and seven-day reuse protocols, respectively. This result contrasts with the findings of a study conducted during the COVID-19 pandemic in three Latin American countries (Brazil, Colombia, and Ecuador), which found a greater provision of training in private institutions than in public ones (p<0.001)^(24)^. This difference may be associated with the profile of the public institution participating in this study, which is involved in teaching and research, which may have favored access to training. Thus, it is observed that, more than the administrative nature (public or private), the institutional and academic context plays a decisive role in training professionals for safe N95 mask use.

It is worth noting that training opportunities remain limited in many contexts. A study conducted in Latin America revealed that 51.4% of healthcare workers had not received training on the proper use of PPE^(20)^. A similar situation was observed in Ethiopia, where less than half of professionals were trained in personal protective measures^(25,26)^.

These data highlight a persistent weakness in healthcare professional training, especially regarding the correct use of PPE, such as the N95 mask. This lack of training is not limited to health crises like SARS-CoV-2, but has also been observed in other public health risk situations, such as those caused by SARS, H1N1, MERS, tuberculosis, and seasonal flu^(27)^. In this context, research confirms that specific training on N95 mask use is essential to significantly reduce the risk of contamination by respiratory pathogens, promoting greater occupational safety^(16,28,29)^.

Another critical point is that most of the training was conducted in 2020 as an emergency response to the COVID-19 pandemic, without continuation in subsequent years. International and national health organizations recommend that these trainings be held regularly, preferably annually, to ensure teams are kept up to date on safe N95 mask use^(2,3,30)^.

“N95 mask donning and doffing” was the most frequently discussed in the training sessions for both protocols analyzed. This emphasis is supported by the literature, which recognizes the importance of these steps for the safety of both healthcare professionals and patients, considering that improper handling of the device can promote cross-contamination and increase the risk of infection^(2,3,12)^. Professionals who received training during the COVID-19 pandemic were 2.4 times more likely to correctly don and undo the N95 mask (OR=2.4; 95%CI: 1.9-3.2)^(29)^. Furthermore, 81.3% of untrained individuals reported difficulties, especially in removing N95 masks, while 66.4% of trained individuals demonstrated greater skill in this step (p<0.001)^(28)^.

Another relevant topic is “hand hygiene”, considered an essential pillar in the prevention and control of the spread of respiratory infectious agents, including, mainly, viruses such as SARS-CoV-2, influenza and respiratory syncytial virus, which can spread through respiratory droplets and contact with contaminated surfaces^(1-3)^. In the present study, this content was covered in approximately 90% of the training sessions in the seven-day protocol, but only in 70% in the 15-day protocol, indicating a worrying reduction in the emphasis given to this preventive practice.

The systematic inclusion of hand hygiene in training is even more critical in N95 mask reuse protocols, where the risk of cross-contamination is increased. This practice should be performed before and after donning, including visual inspection and seal verification, after inadvertent contact with the outside of the N95 mask, and during storage or disposal^(2,3)^. However, evidence points to low adherence among healthcare professionals to this basic prevention measure, which reinforces the need for continuing education programs that consolidate safe behaviors in clinical settings^(31-33)^.

In addition to the widely covered topics, such as donning and doffing, the study revealed that equally crucial topics for safe N95 mask use received less emphasis in training, such as visual inspection, seal verification, and criteria for proper disposal and storage. The absence or superficial approach to these topics can compromise the effectiveness of respiratory protection and increase the risk of occupational exposure^(2,3)^.

Verifying the seal by exhaling and inhaling, although recommended immediately after each dressing and whenever adjustments are needed during extended use, showed low adherence among nursing professionals in both protocols assessed. Less than 20% reported having performed this practice systematically, with lack of knowledge being more significant in the seven-day protocol (13.7%). These findings are consistent with previous studies, which found that among healthcare professionals who reused N95 masks to care for COVID-19 patients, 29% never performed a seal verification or were unaware of its purpose, and 14% performed it occasionally^(34)^. Similarly, another survey revealed that 57% of nurses did not include this step in their routine^(35)^.

Visual inspection of N95 masks before each use was also low: less than half of nursing professionals reported performing it systematically. Although a simple measure, this practice is essential for ensuring occupational safety, as it allows early identification of flaws that compromise the seal on the user’s face and, consequently, the effectiveness of respiratory protection^(2,3)^.

Research conducted in healthcare settings reinforces the importance of visual inspection, especially in contexts of extended use and reuse. In clinical practice, it was observed that, after multiple shifts and frequent donning, N95 masks showed soiling on the inner and outer layers, sharp creases, broken or loosened fastening straps, and damaged nose clips^(34,36-38)^. In a study that analyzed N95 masks used according to reuse protocols for seven and 15 consecutive shifts, damage to the structure and fastening components was identified in both cases, with more pronounced wear in the 15-shift case^(22)^.

In this scenario, the importance of adhering to seal verification and visual inspection of N95 masks becomes evident. Evidence shows that trained professionals adopt these practices more frequently, reducing the risk of occupational exposure^(25,39,40)^.

The effects of training were also analyzed in this study. A significant association was found between training completion and seal verification in the seven-day protocol (p=0.002), indicating greater adherence among trained nursing professionals. However, no statistical differences were observed regarding visual inspection in the two protocols, which may indicate that the impact of training may be limited or not sustainable over time.

This gap may be related to the low frequency of training and the superficiality of the content covered. Approximately 95% of the training sessions took place at the beginning of the pandemic and were not continued in subsequent years. Furthermore, fundamental topics, such as seal verification and visual inspection, were not covered in all training sessions. These findings reinforce the need for continuing education programs, with periodic refresher courses and pedagogical approaches that comprehensively integrate the various aspects of safe N95 mask use, promoting a reflective educational process focused on transforming professional practices^(2,3,21,28)^.

One barrier identified was the lack of dedicated storage spaces for N95 masks in ICUs. The lack of adequate facilities led nursing professionals to adopt improvised strategies, such as storing the devices in backpacks, bags, or individual lockers. These practices can damage the PPE’s structure and fastening components, leading to seal failures and, consequently, occupational risks faced by healthcare professionals by exposing them to infectious agents^(3)^.

Additionally, using plastic bags as an alternative storage method has been reported. However, this type of packaging can promote humidity retention, creating an environment conducive to the proliferation of microorganisms and increasing the risk of cross-contamination in the workplace^(12)^. To ensure proper preservation of N95 masks and the safety of professionals, it is recommended that they be stored in clean, dry, and ventilated environments with controlled temperatures. In these cases, the use of brown paper envelopes or porous plastic bags is recommended as a safe and low-cost alternative^(2,3)^.

### Study limitations

This study has limitations that should be considered. The cross-sectional design prevents the establishment of causal relationships, since the variables were measured simultaneously. Convenience sampling may also limit the generalizability of results. Furthermore, as the data were collected through a questionnaire, there is potential for response bias or underreporting of inappropriate practices. However, conducting the research in two hospital settings with distinct reuse protocols broadens the diversity of the contexts analyzed, strengthening the representativeness of the findings within the studied setting.

### Contributions to nursing

This study expands understanding of the challenges faced by nurses in safe N95 mask reuse in ICUs. By highlighting gaps in training, adherence to safe practices, and the lack of adequate infrastructure for storing N95 masks, the results support the implementation of more effective educational initiatives and institutional adaptations that promote safer care environments aligned with best respiratory protection practices.

## CONCLUSIONS

The findings reveal weaknesses in the perception of safety and the effectiveness of training offered to nursing professionals in ICUs regarding N95 mask reuse protocols. Most participants reported feeling unsafe with the established protocols, citing concerns about humidity, dirt, and a lack of adequate storage space.

Although some professionals reported participating in training, especially the 15-day protocol, these trainings were largely focused on the COVID-19 pandemic and did not consistently cover topics essential for safe N95 mask use. Consequently, adherence to practices such as visual inspection and seal verification was limited, even among those who received training. Only seal verification showed a significant association with training in the seven-day protocol, indicating that training alone did not guarantee the adoption of safe N95 mask use in reuse settings.

The lack of even minimal infrastructure for adequate storage of N95 masks further exacerbates occupational risks. Given this scenario, the need to review institutional reuse protocols, expand the provision of periodic and qualified training, and invest in structural conditions that ensure effective protection for nursing professionals in critical care settings is reinforced.

## Data Availability

The research data are available within the article.
