# Supplementary material for: Nursing perception and practices in N95 mask reuse in intensive care: a cross-sectional study
Source: Rev Bras Enferm. 2026 Jun 22;79:e20250261. doi: 10.1590/0034-7167-2025-0261 (PMC13317008; doi:10.1590/0034-7167-2025-0261)
Supplement: Translation

# Translation: Percepção e práticas de enfermagem no reuso da máscara N95 em terapia intensiva: estudo transversal

**DOI:** 10.1590/0034-7167-2025-0261pt

**Authors:** Viviane Lopes Vimieiro, Claysson Bruno Santos Vimieiro, Adriana Cristina Oliveira

**Affiliations:** I Universidade Federal de Minas Gerais. Belo Horizonte, Minas Gerais, Brasil; II Pontifícia Universidade Católica de Minas Gerais. Belo Horizonte, Minas Gerais, Brasil

**Keywords:** Respiradores N95, Pessoal de Saúde, Saúde Ocupacional, Unidade de Terapia Intensiva, Enfermagem.

## Abstract

**Objetivos:**

analisar a percepção e as práticas dos profissionais de enfermagem de Unidades de Terapia Intensiva quanto ao uso seguro da máscara N95 em protocolos de reuso.

**Métodos:**

estudo transversal realizado em Unidades de Terapia Intensiva.

**Resultados:**

participaram 265 profissionais. A maioria não se sentia segura com o protocolo adotado. As principais preocupações foram umidade e sujidade e armazenamento. Menos de 1% mencionou eficiência de filtração ou capacitação. Receberam treinamento 53,6% (sete dias) e 74,1% (15 dias), principalmente em 2020. Paramentação e desparamentação foi o tema mais abordado. Verificação da vedação e inspeção visual foram sempre realizadas por 20,9% e 46,4% (sete dias), e por 26,8% e 35,7% (15 dias), respectivamente. Houve associação significativa entre treinamento e verificação da vedação no de sete dias. Todos relataram ausência de local exclusivo para armazenamento das máscaras N95.

**Conclusões:**

a percepção de segurança foi frágil, e a capacitação, insuficiente para o reuso seguro.

## INTRODUÇÃO

O uso de equipamentos de proteção individual (EPIs) constitui uma medida essencial para a segurança ocupacional dos profissionais de saúde^(1)^. Entre esses dispositivos, a máscara N95 destaca-se como uma das principais barreiras contra microrganismos respiratórios, como *Mycobacterium tuberculosis*, o vírus da influenza e o coronavírus da síndrome respiratória aguda grave 2 (*Severe Acute Respiratory Syndrome Coronavirus 2* - SARS-CoV-2), agente causador da doença do coronavírus 2019 (COVID-19)^(1-3)^.

Eventos epidemiológicos de grande impacto, como a epidemia de SARS, em 2003, a pandemia de influenza H1N1, em 2009, a emergência do coronavírus da síndrome respiratória do Oriente Médio (*Middle East Respiratory Syndrome Coronavirus* - MERS-CoV), em 2012, e mais recentemente, a pandemia de COVID-19, em 2020, evidenciaram a vulnerabilidade dos sistemas de saúde frente às crises sanitárias globais. Tais contextos resultaram em escassez mundial de máscaras N95, impulsionando a adoção de estratégias alternativas, como o uso estendido e a reutilização desses dispositivos^(4-7)^.

As máscaras N95 são projetadas para garantir uma eficiência mínima de filtração de 95% para partículas de até 0,3 micrômetros (µm), além de assegurar uma vedação facial adequada ao rosto do usuário^(2,3)^. A fabricação e a certificação desses dispositivos são regulamentadas por normas nacionais e internacionais, como a Associação Brasileira de Normas Técnicas (ABNT) NBR 13698:2011, no Brasil, a *European Standard* (EN) 149:2001+A1:2009, na Europa, e o *Code of Federal Regulations* (CFR) 42 Part 84 do *National Institute for Occupational Safety and Health*, nos Estados Unidos, que estabelecem critérios de desempenho e segurança^(8-10)^.

Em instituições de saúde, especialmente aquelas com alta demanda por EPIs, como as Unidades de Terapia Intensiva (UTIs), a reutilização desses dispositivos tem sido adotada como estratégia institucional de racionalização de recursos. Embora funcional em termos de fornecimento, essa prática impõe desafios importantes à proteção do trabalhador. O reuso favorece a degradação estrutural do dispositivo, com danos potenciais à vedação facial devido ao desgaste das tiras de fixação e deformações no clipe nasal^(11,12)^. Além disso, a ausência de locais adequados para armazenamento representa um fator crítico, pois pode contribuir para a contaminação cruzada e a perda da integridade da máscara N95^(11,12)^.

Nesse contexto, os profissionais de enfermagem se destacam por constituírem o maior contingente da equipe multiprofissional e por permanecerem longos períodos em contato direto com pacientes. Estão, portanto, entre os mais vulneráveis à exposição a agentes respiratórios^(13-15)^. Cabe a esses profissionais aplicar corretamente os protocolos institucionais e adotar práticas seguras no manuseio da máscara N95, como inspeção visual, verificação da vedação por meio da expiração e inspiração e armazenamento adequado. Evidências indicam que a adesão a essas condutas está associada ao acesso a capacitações específicas e atualizadas que abordem conteúdos essenciais, como paramentação e desparamentação, verificação da vedação, avaliação da integridade da máscara N95, higienização das mãos, critérios para descarte, e formas apropriadas de armazenamento^(2,3,16)^.

Apesar da relevância do tema, ainda são escassas as evidências que explorem, de forma integrada, a percepção e as práticas dos profissionais de enfermagem diante dos protocolos de reuso da máscara N95, especialmente em contextos de alta complexidade assistencial, como as UTIs. Torna-se, portanto, fundamental compreender como esses profissionais avaliam os protocolos, quais práticas adotam diante das orientações recebidas e de que forma os treinamentos influenciam a adesão às medidas seguras. Essa análise contribui para subsidiar o aperfeiçoamento das estratégias institucionais, fortalecer uma cultura de segurança baseada em evidências e garantir condições adequadas de proteção nos ambientes críticos de cuidado.

## OBJETIVOS

Analisar a percepção e as práticas dos profissionais de enfermagem de UTIs quanto ao uso seguro da máscara N95 em protocolos de reuso.

## MÉTODOS

### Aspectos éticos

O estudo foi conduzido de acordo com os princípios éticos da Resolução n^o^ 466/2012 do Conselho Nacional de Saúde referentes à pesquisa com seres humanos, sendo aprovado pelo Comitê de Ética em Pesquisa da Universidade Federal de Minas Gerais, cujo parecer está anexado à presente submissão.

### Desenho, período e local do estudo

Trata-se de estudo transversal, desenvolvido por meio de entrevistas com profissionais de enfermagem, utilizando um questionário estruturado e norteado pela ferramenta *Strengthening the Reporting of OBservational studies in Epidemiology* ^(17)^. A coleta de dados ocorreu no período de dezembro de 2023 a agosto de 2024 em UTIs adulto de dois hospitais localizados em Belo Horizonte, Minas Gerais, Brasil. Para preservar o anonimato, cada unidade foi identificada com os códigos UA, UB, UC e UD. As UTIs UA e UB pertencem a uma instituição privada, e atende pacientes particulares ou conveniados com 40 leitos de UTI geral e 20 leitos de UTI cardiovascular. As UTIs UC e UD integram uma instituição universitária pública, que atende exclusivamente usuários do Sistema Único de Saúde, possuindo 18 leitos de terapia intensiva e 19 leitos de unidade coronariana.

### População ou amostra; critérios de inclusão e exclusão

A amostra foi selecionada por conveniência e composta por profissionais de enfermagem que atuavam diretamente na assistência nas UTIs selecionadas, utilizando máscaras N95 em protocolos de reutilização com intervalos de sete ou 15 dias. Conforme o protocolo institucional, a substituição podia ocorrer antes do prazo estabelecido em casos de sujidade, umidade, dano estrutural, ruptura das tiras de fixação ou do clipe nasal, perda de vedação ou contaminação por sangue, fluidos corporais, e secreções respiratórias/nasais. Foram excluídos os profissionais de enfermagem que não seguiram esses intervalos, estavam de férias, licença médica ou maternidade ou que se recusaram a responder ao questionário.

O cálculo amostral foi realizado com base no método de estimativa de proporções para população finita, considerando 377 profissionais de enfermagem elegíveis. Adotaram-se uma proporção estimada de 50% (p=0,5), margem de erro de 5% e nível de confiança de 95%, resultando em uma amostra mínima de 191 participantes. No entanto, a amostra final obtida foi composta por 265 profissionais.

### Protocolo do estudo

Os dados foram coletados por meio de questionário estruturado, elaborado pela pesquisadora com base nas diretrizes do *Center for Disease Control and Prevention* e da Agência Nacional de Vigilância Sanitária. O instrumento continha 24 questões organizadas em três seções: (i) variáveis sociodemográficas, laborais e de formação; (ii) aspectos relacionados ao protocolo institucional de reutilização das máscaras N95 e à percepção dos profissionais quanto à segurança proporcionada. Nessa seção, os participantes também indicaram os aspectos considerados fundamentais para garantir a proteção respiratória em protocolos de reuso, como integridade dos itens de fixação, vedação adequada, presença de umidade ou sujidade, risco de contaminação, treinamentos recebidos, condições de armazenamento, e durabilidade e eficiência de filtração; e (iii) participação em treinamento, além da prática de verificação da vedação, inspeção visual e armazenamento das máscaras N95.

Foi realizado teste piloto com profissionais de enfermagem de UTI com perfil semelhante ao das instituições do estudo, com o intuito de avaliar a clareza das perguntas e a aplicabilidade do instrumento. Com base nas observações dessa etapa, pequenos ajustes foram realizados no questionário.

Os profissionais de enfermagem elegíveis foram convidados a participar do estudo e informados sobre os objetivos da pesquisa, o caráter voluntário da participação e a confidencialidade dos dados. Após a leitura e assinatura do Termo de Consentimento Livre e Esclarecido, as entrevistas foram conduzidas presencialmente pelo pesquisador, de forma individual e em horários compatíveis com a rotina de trabalho dos participantes. O tempo médio para preenchimento do questionário foi de aproximadamente dez minutos.

### Análise dos resultados e estatística

Os dados coletados foram armazenados no programa *Microsoft Excel* ^®^ 2019 e, posteriormente, analisados no programa *Statistical Package for the Social Science*, versão 28.0. Na análise descritiva, as variáveis qualitativas foram descritas por frequências absolutas e relativas. Para as variáveis quantitativas, recorreu-se à média, ao desvio padrão e às medidas de amplitude. A fim de comparar a relação entre a participação em treinamento e a frequência com que os profissionais de enfermagem adotavam práticas de segurança ao utilizar máscaras N95, especificamente em termos de verificação da vedação e inspeção visual, utilizaram-se os teste qui-quadrado e exato de Fisher. O nível de significância estabelecido em todas as análises foi de 5%, com um Intervalo de Confiança de 95%.

## RESULTADOS

Foram elegíveis para o estudo 377 profissionais de enfermagem. Nas UTIs com protocolo de reuso de sete dias, 74,3% (n=153) dos 206 elegíveis participaram; 8,7% (n=18) foram excluídos por afastamento (férias, licenças médicas ou maternidade); e 17% (n=35) recusaram. Nas UTIs com protocolo de 15 dias, participaram 65,5% (n=112) dos 171 elegíveis; 9,4% (n=16) foram excluídos por afastamentos; e 25,1% (n=43) recusaram. Portanto, a amostra foi constituída por 268 (71,1%) profissionais, cujas características sociodemográficas, laboral e de formação estão apresentadas na Tabela 1.

**Tabela 1:** Características sociodemográficas, laborais e de formação de profissionais de enfermagem das Unidades de Terapia Intensiva que adotaram protocolos de reuso de máscara N95 por sete (n=153) e 15 dias (n=112), Belo Horizonte, Minas Gerais, Brasil, 2025

Variáveis	Protocolo de sete dias (n=153)		Protocolo de 15 dias (n=112)	
	n	%	n	%
**Sexo**				
Feminino	116	75,8	81	72,3
Masculino	37	24,2	31	27,7
**Categoria profissional**				
Enfermeiro	25	16,3	68	60,7
Técnico em enfermagem	128	83,7	44	39,3
**Titulação**				
Graduação	10	40,0	8	11,8
Especialista	15	60,0	53	77,9
Mestre	0	0,0	6	8,8
Doutor	0	0,0	1	1,5
**Idade (anos)** Média (±DP/amplitude)	39,4 (±8,9; 22-61)		40,8 (±6,6; 25-57)	
**Tempo de formação profissional (anos)** Média (±DP/amplitude)	13,4 (±7,5; 2-34		15,5 (±5,8; 2-26)	
**Tempo de atuação profissional (anos)** Média (±DP/amplitude)	13,2 (±7,5; 2-34)		15,2 (±6,0; 2-26)	
**Tempo de atuação na instituição (anos)**				
Média (±DP/amplitude)	8,1 (±6,2; 1-34)		7,3 (±4,6;1-23)	
**Tempo de atuação na UTI (anos)**				
Média (±DP/amplitude)	10,7 (±6,7; 1-34)		8,6 (±5,1; 1-24)	
**Turno de trabalho**				
Diurno	81	52,9	64	57,1
**Carga horária trabalho semanal (horas)**				
≤36	19	12,4	104	92,9
≥40	134	87,6	8	7,1
**Atuação em outra instituição**				
Sim	44	28,8	42	37,5
Não	109	71,2	70	62,5

*DP - Desvio Padrão; UTI - Unidade de Terapia Intensiva.*

Observou-se predomínio de profissionais de enfermagem do sexo feminino em ambos os protocolos. No protocolo de sete dias, a maioria era técnico de enfermagem (n=128; 83,7%), enquanto que, no de 15 dias, prevaleceram enfermeiros (n=68; 60,7%). A titulação também diferiu entre os grupos. No protocolo de 15 dias, houve maior percentual de especialistas quanto à titulação (n=53; 77,9%), sendo mestres (n=6; 8,8%) e doutores (n=1; 1,5%), enquanto que, no protocolo de sete dias, a maioria possuía apenas graduação (n=10; 40,0%) ou especialização (n=15; 60,0%).

Duas UTIs adotaram protocolo de reutilização de máscaras N95 por até sete plantões consecutivos, e outras duas, por até 15 plantões, com turnos de seis a 12 horas. Quanto à percepção dos profissionais de enfermagem sobre a segurança desses protocolos, 42,5% (n=65) dos participantes vinculados ao protocolo de sete dias o consideraram seguro; 25,5% (n=39) o classificaram como inseguro; e 32% (n=49) não souberam informar. No protocolo de 15 dias, 33% (n=37) consideraram o protocolo seguro; 25,9% (n=29) o julgaram inseguro; e 41,1% (n=46) não souberam informar.

Adicionalmente, a Figura 1 apresenta os aspectos abordados pelos profissionais de enfermagem como fundamentais para garantir a proteção respiratória durante os protocolos de reuso das máscaras N95.

**Figura 1:** Proteção respiratória percebida pelos profissionais de enfermagem de Unidades de Terapia Intensiva com protocolos de reuso de máscara N95 por sete (n=153) e 15 dias (n=112), Belo Horizonte, Minas Gerais, Brasil, 2025

Na análise do protocolo de reutilização da máscara N95 por sete e 15 dias, aspectos como “umidade e sujidade” e “condições seguras de armazenamento” se destacaram como pontos de atenção entre os profissionais de enfermagem. Em contrapartida, a “garantia da capacidade mínima de filtração” foi mencionada por apenas um profissional (0,9%) em ambos os protocolos, enquanto a “realização de treinamentos” apareceu apenas no protocolo de 15 dias, também citada por um único participante. Esses dados indicam que, mesmo com a possibilidade de múltiplas respostas na questão aberta, esses critérios receberam pouca ênfase.

No que diz respeito à participação em treinamentos sobre o uso da máscara N95, 53,6% (n=82) dos profissionais de enfermagem do protocolo de sete dias relataram ter recebido capacitação, enquanto que, no protocolo de 15 dias, esse percentual foi de 74,1% (n=83). Para a maioria, os treinamentos ocorreram durante a pandemia de COVID-19: 79,3% (n=65) no grupo de sete dias e 88% (n=73) no de 15 dias. Em ambos os protocolos, a duração foi inferior a duas horas e, predominantemente, as capacitações ocorreram na própria instituição. A Tabela 2 detalha os dados.

**Tabela 2:** Participação dos profissionais de enfermagem de Unidades de Terapia Intensiva com protocolos de reuso de sete dias (n=153) e 15 dias (n=112) em treinamentos sobre o uso da máscara N95, Belo Horizonte, Minas Gerais, Brasil, 2025

Variáveis	Protocolo de sete dias (n=153)		Protocolo de 15 dias (n=112)	
	n	%	n	%
**Participação treinamento**				
Sim	82	53,6	83	74,1
Não	66	43,1	26	23,2
Não sei	5	3,3	3	2,7
**Ano**				
2020	65	79,3	73	88,0
2020 e 2021	5	6,1	3	3,6
2021 2022 2023	8 3 1	9,7 3,7 1,2	4 2 1	4,8 2,4 1,2
**Duração**				
Menos de duas horas	69	84,2	77	98,2
Mais de duas horas	12	14,6	3	3,6
Não sabe informar	1	1,2	3	3,6
**Local**				
Na própria instituição	70	85,4	61	73,5
Em outra instituição	3	3,6	10	12,0
Em ambos os locais	9	11,0	12	14,5

No contexto dos conteúdos abordados nos treinamentos sobre o uso de máscaras N95, a Figura 2 detalha os temas abordados durante as capacitações.

**Figura 2:** Conteúdos abordados no treinamento para profissionais de enfermagem das Unidades de Terapia Intensiva com protocolos de reuso de sete (n=153) e 15 dias (n=112) sobre o uso da máscara N95, Belo Horizonte, Minas Gerais, Brasil, 2025

“Paramentação e desparamentação” foi o tema mais abordado, em ambos os protocolos, por 77 (92,8%) profissionais, no protocolo de 15 dias, e 75 (91,5%) profissionais, no protocolo de sete dias. No protocolo de sete dias, destacaram-se “uso correto e seguro” (72; 87,8%) e “higienização das mãos” (74; 90,2%). Já os menos abordados foram “avaliação da integridade” (54; 65,1%) e “verificação da vedação” (56; 67,4%), no de 15 dias, e “armazenamento seguro” (58; 70,7%) e “critérios para descarte” (60; 73,2%), no de sete dias.

Quanto à adoção das práticas seguras, no protocolo de sete dias, a verificação da vedação era realizada raramente, por 45,8% (n=70), sempre, por 20,9% (n=32), nunca, por 19,6% (n=30), e era desconhecida por 13,7% (n=21). No protocolo de 15 dias, os percentuais foram 50,0% (n=56) relataram realizar a prática raramente, 26,8% (n=30), sempre, 17,9% (n=20), nunca, e 5,4% (n=6) desconheciam essa etapa. Sobre a inspeção visual, no protocolo de sete dias, 47,7% (n=73) relataram fazê-la raramente, 46,4% (n=71), sempre, e 5,9% (n=9), nunca, e no protocolo de 15 dias, 60,7% (n=68) a realizavam raramente, 35,7% (n=40), sempre, e 3,6% (n=4), nunca. A Tabela 3 apresenta a associação entre participação em treinamentos e adoção de práticas relacionadas à verificação da vedação e à inspeção visual das máscaras N95.

**Tabela 3:** Treinamento sobre o uso da máscara N95 e práticas de verificação da vedação e a inspeção visual entre profissionais de enfermagem em Unidades de Terapia Intensiva com protocolos de reuso por sete dias (n=153) e 15 dias (n=112), Belo Horizonte, Minas Gerais, Brasil, 2025

Práticas	Variáveis	Treinamentos				Valor de *p*
		Sim (n=82)		Não (n=66)		
		n	%	n	%	
**Protocolo de sete dias**						
**Verificação da vedação**	Desconhece	6	7,3	15	22,7	0,002^*^
	Nunca	13	15,9	17	25,8	
	Raramente	44	53,6	21	31,8	
	Sempre	19	23,2	13	19,7	
**Inspeção visual**	Nunca	3	3,7	6	9,1	0,198^**^
	Raramente	35	42,6	37	56,1	
	Sempre	44	53,7	23	34,8	
**Práticas**	**Variáveis**	**Treinamentos**				**Valor de *p***
		**Sim (n=83)**		**Não (n=29)**		
		**n**	**%**	**n**	**%**	
**Protocolo de 15 dias**						
**Verificação da vedação**	Desconhece	3	3,6	3	10,3	0,374^**^
	Nunca	15	18,1	5	17,2	
	Raramente	42	50,6	14	48,3	
	Sempre	23	27,7	7	24,1	
**Inspeção visual**	Nunca	2	2,4	2	6.9	0,298^**^
	Raramente	54	65,1	14	48,3	
	Sempre	27	32,5	13	44,8	

* teste qui-quadrado;

** teste exato de Fisher.

A comparação entre profissionais treinados e não treinados revelou diferença estatisticamente significativa na verificação da vedação apenas no protocolo de sete dias (p=0,002), com maior adesão entre os que participaram de treinamentos. Para a inspeção visual, não foram observadas diferenças estatísticas relevantes em nenhum dos protocolos, indicando que o treinamento não influenciou a adoção dessa prática.

No que se refere ao local de armazenamento, todos os participantes de ambos os protocolos (100%, n=153; n=112) relataram ausência de um espaço exclusivo para armazenar as máscaras N95 após o uso nas instituições onde trabalhavam. Diante dessa limitação, os participantes adotaram diferentes estratégias de acondicionamento. No protocolo de sete dias, a maioria armazenava as máscaras em sacos de plástico dentro de bolsas ou armários pessoais (61,4%; n=94). Já no protocolo de 15 dias, o armário pessoal foi o local preferencial, sendo adotado por 43,8% (n=49), enquanto o uso de saco de plástico foi menor (6,3%; n=7).

## DISCUSSÃO

Os achados deste estudo evidenciam aspectos críticos relacionados à percepção e às práticas dos profissionais de enfermagem de UTIs quanto ao uso seguro da máscara N95 em protocolos de reuso. Nessas instituições, é permitida a reutilização por sete ou 15 plantões consecutivos, em turnos de seis ou 12 horas, desde que as máscaras N95 se mantenham limpas, íntegras, secas e com vedação facial adequada. A continuidade dessa medida, mesmo após o fim da emergência de saúde pública global causada pela COVID-19, reflete sua consolidação como estratégia institucional para redução de custos.

Contudo, essas diretrizes contrastam com as recomendações de órgãos reguladores internacionais e nacionais, que classificam as máscaras N95 como dispositivos de uso único, recomendando seu descarte após cada utilização^(2,3)^. Normas técnicas de diferentes países estabelecem parâmetros rigorosos de desempenho e eficiência de filtração, mas não contemplam critérios para reutilização, evidenciando uma lacuna regulatória^(8-10)^. Além disso, observa-se variabilidade entre países e instituições: algumas normativas permitem apenas o uso estendido ou a reutilização limitada, enquanto outras combinam ambas as práticas, resultando em protocolos distintos^(6,18-20)^.

Essa heterogeneidade evidencia a ausência de consenso sobre os limites seguros de reutilização das máscaras N95, o que pode comprometer a confiança dos profissionais de enfermagem nos protocolos adotados. Neste estudo, a maioria dos participantes relatou não considerar seguro o protocolo de sete dias (71,9%) ou o de 15 dias (60,7%), sinalizando uma percepção de insegurança mesmo na presença de protocolos definidos localmente. Esses achados corroboram a literatura, que aponta que a falta de protocolos claros e unificados contribui para a insegurança dos trabalhadores de saúde, destacando a necessidade de diretrizes consistentes e embasadas em evidências para garantir o uso seguro da máscara N95 em ambientes de saúde^(21)^.

Os profissionais de enfermagem identificaram como principais fatores de riscos à proteção respiratória, nos protocolos de reuso da máscara N95 de sete e 15 dias, a presença de umidade e sujidade, e o armazenamento inadequado. Esses aspectos refletem percepções de práticas relevantes da equipe, pois comprometem a integridade estrutural das máscaras N95 ao longo do tempo de uso. Durante a reutilização, máscaras N95 tendem a apresentar acúmulo de sujidade nas camadas externas e internas, além de retenção de umidade, condições agravadas pela inexistência de local adequado para acondicionamento. Esses fatores, somados, contribuem para o desgaste do dispositivo, e podem aumentar o risco ocupacional dos profissionais de saúde^(22)^.

Entretanto, aspectos igualmente essenciais para a segurança ocupacional nos protocolos de reuso, como a manutenção da eficiência de filtração e a realização de treinamentos, foram mencionados por menos de 1% dos participantes. Essa baixa frequência revela uma lacuna importante na percepção sobre critérios fundamentais quanto à capacidade do dispositivo em manter sua eficácia ao longo do tempo. Pesquisa quase-experimental demonstrou que, após o uso por 14 plantões consecutivos, a eficiência de filtração das máscaras N95 foi reduzida para 80,4%, comprometendo a proteção oferecida ao usuário^(23)^. Além disso, a pouca valorização dos treinamentos sugere a necessidade de reforçar, nos processos formativos, a importância da capacitação contínua como estratégia para o uso seguro do EPI.

Quanto à participação em treinamentos sobre o uso da máscara N95, verificou-se maior proporção de profissionais capacitados na instituição pública (74,1%), em comparação à privada (53,6%), nos protocolos de reuso de 15 e sete dias, respectivamente. Esse resultado contrasta com os achados de pesquisa realizada durante a pandemia de COVID-19 em três países da América Latina (Brasil, Colômbia e Equador), que identificou maior oferta de capacitações em instituições privadas do que nas públicas (p<0,001)^(24)^. Essa diferença pode estar associada ao perfil da instituição pública participante deste estudo, vinculada ao ensino e à pesquisa, o que pode ter favorecido o acesso à capacitação. Dessa forma, observa-se que, mais do que a natureza administrativa (pública ou privada), o contexto institucional e acadêmico desempenha um papel determinante na formação dos profissionais para o uso seguro das máscaras N95.

Vale destacar que a oferta de treinamentos ainda é limitada em diversos contextos. Estudo conduzido na América Latina revelou que 51,4% dos trabalhadores de saúde não tinham recebido capacitação sobre o uso adequado de EPIs^(20)^. Situação semelhante foi observada na Etiópia, onde menos da metade dos profissionais foi capacitada em medidas de proteção individual^(25,26)^.

Esses dados evidenciam uma fragilidade persistente na formação dos profissionais de saúde, especialmente no que diz respeito ao uso correto de EPIs, como a máscara N95. A carência de capacitação não é um fenômeno restrito às crises sanitárias, como a do SARS-CoV-2, mas também foi observada em outras situações de risco à saúde pública, como as causadas por SARS, H1N1, MERS, tuberculose e gripe sazonal^(27)^. Nesse contexto, pesquisas confirmam que treinamentos específicos sobre o uso de máscaras N95 são essenciais para reduzir significativamente o risco de contaminação por patógenos respiratórios, promovendo maior segurança ocupacional^(16,28,29)^.

Outro ponto crítico é que a maioria dos treinamentos foi realizada em 2020, como resposta emergencial à pandemia COVID-19, sem continuidade nos anos subsequentes. Organizações internacionais e nacionais de saúde recomendam que essas capacitações sejam regulares, preferencialmente anuais, para garantir a atualização constante das equipes quanto ao uso seguro da máscara N95^(2,3,30)^.

O tema “paramentação e desparamentação das máscaras N95” foi o mais abordado nos treinamentos em ambos os protocolos analisados. Essa ênfase é respaldada na literatura, que reconhece a importância dessas etapas na segurança tanto dos profissionais de saúde quanto dos pacientes, considerando que a manipulação inadequada do dispositivo pode favorecer a contaminação cruzada e o aumento do risco de infecções^(2,3,12)^. Profissionais que receberam treinamento durante a pandemia de COVID-19 apresentaram 2,4 vezes mais chances de realizar corretamente a paramentação e desparamentação da máscara N95 (OR=2,4; IC95%: 1,9-3,2)^(29)^. Ademais, 81,3% dos indivíduos sem capacitação relataram dificuldades, especialmente na remoção das máscaras N95, enquanto 66,4% dos capacitados demonstraram maior habilidade nessa etapa (p<0,001)^(28)^.

Outro tema relevante é a “higienização das mãos”, considerada um pilar essencial na prevenção e no controle da disseminação de agentes infecciosos respiratórios, incluindo, principalmente, vírus como o SARS-CoV-2, influenza e o vírus sincicial respiratório, que podem se propagar por gotículas respiratórias e contato com superfícies contaminadas^(1-3)^. No presente estudo, esse conteúdo foi abordado em cerca de 90% das capacitações no protocolo de sete dias, mas apenas em 70% no protocolo de 15 dias, indicando uma redução preocupante na ênfase atribuída a essa prática preventiva.

A inclusão sistemática da higienização das mãos nos treinamentos é ainda mais crítica em protocolos de reuso da máscara N95, no qual o risco de contaminação cruzada é ampliado. Tal prática deve ser realizada antes e após a paramentação, incluindo os momentos de inspeção visual e verificação da vedação, após contato inadvertido com a parte externa da máscara N95 e nos momentos de armazenamento ou descarte^(2,3)^. No entanto, evidências apontam para baixa adesão dos profissionais de saúde a essa medida básica de prevenção, o que reforça a necessidade de programas de educação permanente que consolidem comportamentos seguros no ambiente clínico^(31-33)^.

Além dos temas amplamente abordados, como paramentação e desparamentação, o estudo revelou que tópicos igualmente cruciais para o uso seguro da máscara N95 receberam menos ênfase nos treinamentos, como inspeção visual, verificação da vedação e critérios para descarte e armazenamento adequado. A ausência ou abordagem superficial desses temas pode comprometer a eficácia da proteção respiratória e aumentar o risco de exposição ocupacional^(2,3)^.

A verificação da vedação por meio da expiração e inspiração, embora recomendada imediatamente após cada paramentação e sempre que houver necessidade de ajuste durante o uso estendido, apresentou baixa adesão entre os profissionais de enfermagem nos dois protocolos avaliados. Menos de 20% afirmaram ter realizado essa prática de forma sistemática, sendo o desconhecimento mais expressivo no protocolo de sete dias (13,7%). Esses achados são consistentes com estudos anteriores, que identificaram que, entre profissionais de saúde que reutilizaram máscaras N95 para atendimento de pacientes com COVID-19, 29% nunca realizaram a verificação da vedação ou desconheciam sua finalidade, e 14% realizavam ocasionalmente^(34)^. De forma semelhante, outra pesquisa revelou que 57% dos enfermeiros não incluíram essa etapa em sua rotina^(35)^.

A inspeção visual das máscaras N95 antes de cada uso também apresentou baixa frequência: menos da metade dos profissionais de enfermagem declarou realizá-la de forma sistemática. Embora seja uma medida simples, essa prática é fundamental para garantir a segurança ocupacional, pois permite a identificação precoce de falhas que comprometem a vedação ao rosto do usuário e, consequentemente, a eficácia da proteção respiratória^(2,3)^.

Pesquisas realizadas em serviços de saúde reforçam a relevância da inspeção visual, especialmente em contextos de uso estendido e reutilização. Na prática clínica, observou-se que, após múltiplos plantões e frequentes paramentações, as máscaras N95 apresentaram sujidade nas camadas internas e externas, dobras acentuadas, rompimento ou afrouxamento das tiras de fixação e danos no clipe nasal^(34,36-38)^. Em estudo que analisou máscaras N95 utilizadas conforme protocolos de reuso de sete e 15 plantões consecutivos, foi identificado comprometimento da estrutura e dos componentes de fixação em ambos os casos, com desgaste mais acentuado no de 15 turnos^(22)^.

Neste cenário, torna-se evidente a importância da adesão à verificação da vedação e à inspeção visual das máscaras N95. Evidências demonstram que profissionais capacitados adotam essas práticas com maior frequência, reduzindo o risco de exposição ocupacional^(25,39,40)^.

Os efeitos dos treinamentos também foram analisados neste estudo. Verificou-se associação significativa entre a realização de capacitação e a verificação da vedação no protocolo de sete dias (p=0,002), indicando maior adesão entre os profissionais de enfermagem capacitados. No entanto, não foram observadas diferenças estatísticas quanto à inspeção visual nos dois protocolos, o que pode sinalizar que o impacto dos treinamentos pode ser limitado ou não sustentável ao longo do tempo.

Essa lacuna pode estar relacionada à baixa frequência de capacitações e à superficialidade dos conteúdos abordados. Cerca de 95% dos treinamentos ocorreram no início da pandemia, sem continuidade nos anos seguintes. Além disso, temas fundamentais, como a verificação da vedação e a inspeção visual, não foram contemplados em todos os treinamentos. Esses achados reforçam a necessidade de programas de educação continuada, com reciclagem periódica e abordagens pedagógicas que integrem, de forma abrangente, os diversos aspectos do uso seguro da máscara N95, promovam um processo educativo reflexivo e voltado à transformação das práticas profissionais^(2,3,21,28)^.

Uma barreira identificada foi a ausência de locais exclusivos para o armazenamento das máscaras N95 nas UTIs. A inexistência de estruturas adequadas levou os profissionais de enfermagem a adotarem estratégias improvisadas, como acondicionar os dispositivos em mochilas, bolsas ou armários individuais. Essas práticas podem danificar a estrutura e os componentes de fixação do EPI, possibilitando falhas na vedação e, consequentemente, os riscos ocupacionais enfrentados pelos profissionais de saúde ao expô-los a agentes infecciosos^(3)^.

Além disso, foi relatado o uso de sacos plásticos como alternativa de armazenamento. No entanto, esse tipo de acondicionamento pode favorecer a retenção de umidade, criando um ambiente propício à proliferação de microrganismos e elevando o risco de contaminação cruzada no ambiente de trabalho^(12)^. Para garantir a conservação adequada das máscaras N95 e a segurança dos profissionais, recomenda-se o armazenamento em ambientes limpos, secos e ventilados com temperatura controlada. Nesses casos, o uso de envelopes de papel pardo ou sacos plásticos porosos é indicado como alternativa segura e de baixo custo^(2,3)^.

### Limitações do estudo

Este estudo apresenta limitações que devem ser consideradas. O delineamento transversal impede o estabelecimento de relações de causalidade, uma vez que as variáveis foram mensuradas simultaneamente. A amostragem por conveniência também pode restringir a generalização dos resultados. Além disso, como os dados foram obtidos por meio de questionário, há possibilidade de viés de resposta ou subnotificação de práticas inadequadas. No entanto, a condução da pesquisa em dois campos de instituições hospitalares com protocolos de reuso distintos amplia a diversidade dos contextos analisados, fortalecendo a representatividade dos achados dentro do cenário estudado.

### Contribuições para a área da enfermagem

Este estudo amplia a compreensão sobre os desafios enfrentados pela enfermagem no reuso seguro da máscara N95 em UTIs. Ao evidenciar falhas nos treinamentos, na adesão a práticas seguras e na ausência de infraestrutura adequada para o armazenamento das máscaras N95, os resultados subsidiam a implementação de ações educativas mais eficazes e adaptações institucionais que promovam ambientes de cuidado mais seguros e alinhados às melhores práticas de proteção respiratória.

## CONCLUSÕES

Os achados revelam fragilidades na percepção de segurança e na efetividade dos treinamentos oferecidos aos profissionais de enfermagem em UTIs diante dos protocolos de reuso da máscara N95. A maioria dos participantes relatou não se sentir segura com os protocolos instituídos, apontando preocupações com umidade, sujidade e falta de locais adequados para armazenamento.

Embora parte dos profissionais tenha relatado participação em treinamentos, especialmente no protocolo de 15 dias, essas capacitações se concentraram majoritariamente durante a pandemia de COVID-19, e não contemplaram, de forma consistente, temas essenciais para o uso seguro da máscara N95. Como consequência, a adesão a práticas como inspeção visual e verificação da vedação foi limitada, mesmo entre os que receberam treinamentos. Apenas a verificação da vedação apresentou associação significativa com a capacitação no protocolo de sete dias, indicando que o treinamento, por si só, não garantiu a adoção do uso seguro das máscaras N95 em contextos de reuso.

A ausência de infraestrutura mínima para o armazenamento adequado das máscaras N95 agrava ainda mais os riscos ocupacionais. Diante desse cenário, reforça-se a necessidade de revisar os protocolos institucionais de reutilização, ampliar a oferta de treinamentos periódicos e qualificados, e investir em condições estruturais que assegurem a proteção efetiva dos profissionais de enfermagem em ambientes críticos de cuidado.

## Data Availability

Os dados de pesquisa estão disponíveis no corpo do artigo.
